# Urinary Podocalyxin as a Biomarker to Diagnose Membranous Nephropathy

**DOI:** 10.1371/journal.pone.0163507

**Published:** 2016-09-26

**Authors:** Takahiro Imaizumi, Masahiro Nakatochi, Shin’ichi Akiyama, Makoto Yamaguchi, Hiroyuki Kurosawa, Yoshiaki Hirayama, Takayuki Katsuno, Naotake Tsuboi, Masanori Hara, Shoichi Maruyama

**Affiliations:** 1 Department of Nephrology, Nagoya University Graduate School of Medicine, Nagoya, Japan; 2 Biostatistics and Bioinformatics Section, Center for Advanced Medicine and Clinical Research, Nagoya University Hospital, Nagoya, Japan; 3 Department of Nephrology, Yokkaichi Municipal Hospital, Yokkaichi, Japan; 4 Denka Co., Ltd., Tokyo, Japan; 5 Department of Pediatrics, Niigata prefecture Yoshida hospital, Tsubame, Japan; Center for Molecular Biotechnology, ITALY

## Abstract

**Background:**

A non-invasive diagnostic marker of membranous nephropathy (MN) is desirable. The urinary level of podocalyxin (PCX) is higher in various glomerular diseases, including MN. The aim of this study was to construct a diagnostic model of MN with the combination of urinary PCX and clinical parameters.

**Methods:**

We performed this cross-sectional study to construct the diagnostic models for MN by using data and samples from the multicenter kidney biopsy registry of Nagoya University and its affiliated hospitals. Urinary (u-) PCX was measured by sandwich ELISA. We constructed 3 types of diagnostic models in 105 training samples: u-PCX univariate model, the combined model of clinical parameters other than u-PCX (clinical model), and the combined model of both u-PCX and clinical parameters (combined model). We assessed the clinical usefulness of the diagnostic models through the comparison of c-statistics and decision curve analysis (DCA) in 209 validation samples.

**Results:**

The clinical model consisted of age, glomerular filtration rate, and diabetes mellitus. In the training cohort, the c-statistics were 0.868 [95% CI, 0.799–0.937] in the combined model. In the validation cohort, sensitivity was 80.5% and specificity was 73.5% on the cut-off value. The net benefit of the combined model was better between threshold probabilities of 40–80% in DCA.

**Conclusions:**

In this study, we demonstrated the utility of u-PCX as a diagnostic marker for MN and the clinical usefulness of the diagnostic models, through the combination of u-PCX and clinical parameters including age, glomerular filtration rate, and diabetes mellitus.

## Introduction

Membranous nephropathy (MN) is one of the major subtypes of nephrotic syndrome (NS), where the clinical course is often chronic and requires long term immunosuppressive therapy[1]. Diagnosis is critical, but it is sometimes difficult to perform kidney biopsy because of comorbidities and treatments such as anti-thrombotic therapy. Thus, a non-invasive diagnostic marker of MN is desirable.

The anti-PLA2R antibody is a well-known biomarker used to diagnose idiopathic MN, and is highly specific but not so sensitive[2]. The sensitivity of anti-PLA2R antibody was reported to be 52%–78%[2–5], but only 53% among the Japanese population[6]. To our knowledge, there are no sensitive screening markers or diagnostic models of MN.

Podocalyxin (PCX) is a transmembrane protein which localizes to the apical cell of glomerular podocytes[7]. PCX functions to maintain podocytes’ shape and slit diaphragm[8]. Hara et al. reported that PCX is shed from injured podocytes into urine, as small vesicles that appear on the tip of glomerular podocyte microvilli[9]. The levels of urinary PCX (u-PCX) increased significantly in patients with diabetic nephropathy and NS[10], or other active glomerulonephritis[11–13]. In a prior study, the level of u-PCX was high in MN, but there were only 9 cases of MN, and no analysis were performed for the significance of u-PCX as a diagnostic marker of MN to differentiate from other glomerular diseases[10].

Traditionally, performance of diagnostic models has been evaluated by comparing the area under the receiver operating characteristic (ROC) curve (AUC). However, AUC alone is not sufficient to show that a model would improve decision-making[14]. Decision curve analysis (DCA), first described by Vickers and Elkin, can be used to incorporate the clinical consequences of a decision into evaluations of diagnostic tests[15].

Here, our objective was to construct and evaluate models by using u-PCX and clinical parameters to diagnose MN. Firstly, we constructed the models in the training cohort of representative kidney disease cases that can cause NS. Secondly, we validated the models in consecutive NS cases. Then, to evaluate the clinical usefulness of diagnostic models, we performed the ROC analysis and DCA.

## Materials and Methods

### Study design

To construct the diagnostic models for MN, we collected data and samples from Nagoya Kidney Disease Registry (N-KDR), the multicenter kidney biopsy registry of Nagoya University and its affiliated hospitals; Handa City Hospital, Tsushima City Hospital, Japanese Red Cross Nagoya Daiichi Hospital, Ichinomiya Municipal Hospital, Kainan Hospital, Ogaki Municipal Hospital, Toyota Kosei Hospital, Yokkaichi Municipal Hospital, Kasugai Municipal Hospital, Anjo Kosei Hospital, Tosei General Hospital, Konan Kosei Hospital, Chubu Rosai Hospital, Toyohashi Municipal Hospital, Chutoen General Medical Center, Tokai Central Hospital, and Nagoya Kyoritsu Hospital.

This study was approved by the ethics committee of Nagoya University Hospital (No. 1135–21), and conducted according to Declaration of Helsinki guidelines. Written Informed consent was obtained from all patients prior kidney biopsy.

First, we specified the training cohort and the validation cohort from N-KDR. We then constructed the diagnostic models by using data in the training cohort, and validated the data in the validation cohort. The diagnostic models contained 3 types of models: Model A, univariate of u-PCX; Model B, combination of clinical parameters other than u-PCX; Model C, the combined model of u-PCX and clinical parameters.

Second, we assessed the ability of the diagnostic models by comparing the area under ROC curve (AUC) between 3 models to discriminate between MN cases and others. In regards to clinical usefulness, we examined net benefit by using DCA.

Finally, we validated each models in the validation cohort by using the same regression equations that were constructed in the training cohort.

### Patients

#### Case definition

NS was defined as serum albumin ≤ 3.0 g/dL and urinary protein level ≥ 3.5 g/day and/or urinary protein/creatinine ratio ≥ 3.5 g/gCr.

MN was proven by kidney biopsy, according to the KDIGO clinical practice guideline for glomerulonephritis[16]. Diagnostic features included capillary wall thickening, normal cellularity, IgG and C3 found along capillary walls upon immunofluorescence, and sub-epithelial deposits visible by electron microscopy. In this study, we included the secondary MN (such as membranous lupus nephritis) in the analysis because we considered u-PCX to be reflective of the pathological changes in any underlying diseases.

#### Training samples

We randomly collected 105 urine samples of representative kidney diseases that can cause NS, from 2009 to 2011. The kidney diseases included minimal change disease (MCD), MN, focal segmental glomerulosclerosis (FSGS), membranoproliferative glomerulonephritis (MPGN), lupus nephritis (LN), diabetic nephropathy (DN), and amyloidosis. LN was classified into 2 subclasses: LN (V) and LN (non-V) according to the 2003 ISN/RPS classification. A Class V lesion was defined as global or segmental sub-epithelial immune deposits or their morphologic sequelae, with or without class III/IV lesion. Data pertaining to baseline characteristics were prospectively collected at kidney biopsy. Urinary samples were collected prior to kidney biopsy.

#### Validation samples

We selected consecutive NS cases between January 2012 and December 2013. To construct the validation cohort, we extracted 358 consecutive NS cases from 1274 kidney biopsies between January 2012 and December 2013. After excluding cases where urine samples were not obtained or those who received immunosuppression treatment at the time of kidney biopsy, 209 NS cases were included. The 149 excluded cases consisted of 54 MCD, 27 were MN, 10 were LN, and others.

### Measurement

#### Clinical information

Data pertaining to baseline characteristics were collected from kidney biopsy registration sheets, which attending doctors sent to Nagoya University. The registration sheets include medical history, concise physical examination, and laboratory data prior to biopsy. Baseline data included age, gender, history of diabetes mellitus, body mass index, systolic blood pressure, microscopic hematuria, serum protein, serum albumin, serum cholesterol, serum creatinine, estimated glomerular filtration rate (eGFR) as described by Matsuo et al.[17], and a positive result for autoantibodies.

#### Diagnosis through kidney biopsy

Immediately after biopsy, specimens were fixed in buffered formalin, sent to Nagoya University, and embedded in paraffin. All samples were observed under light microscopy (PAS, PAM, Masson, hematoxylin eosin stains), immunofluorescence microscopy (IgG, IgA, IgM, C3, C4, C1q, κ, λ), and electron microscopy. Final diagnoses were made in the kidney biopsy pathology conference.

#### Urine sample collection

Urine samples were collected in sterile plastic tubes and mixed with a protease inhibitor mixture (final concentration of 0.01% [w/v] NaN3, 10 mM ε-amonocaproic, 20 mM EDTA, 10 mM Tris, 0.05% [w/v] Tween20, and 10 mM benzamidine). Urine samples were put in the refrigerators soon after collection and sent to the Department of Nephrology in Nagoya University School of Medicine in refrigerated package within a day. Samples were centrifuged at 3000 rpm for 10 min immediately upon arrival, frozen at -80°C and thawed once prior to analysis.

#### Urinary biomarkers

U-PCX was measured by sandwich ELISA as described previously[12]. We measured urinary protein, albumin, α1-microglobulin (AMG), β2-microglobulin (BMG), and N-acetyl-β-D-glucosaminidase (NAG) as existing urinary biomarkers. Urinary creatinine was measured for normalization of urinary markers. Levels of urinary protein excretion were measured by the pyrogallol red method using a reagent kit (Protein Assay Rapid Kit, Wako Pure Chemical Industries, Ltd.). Levels of urinary creatinine were measured by an automated machine (7170S; Hitachi), using a reagent kit (CRE-S; Denka Seiken Co., Ltd.). Levels of urinary albumin were measured by the TIA method (ALB-TIA; Denka Seiken Co., Ltd.). Levels of urinary BMG and urinary AMG were measured by the latex agglutination method (BMG-latex X1, αMi-latex, respectively; Denka Seiken Co., Ltd.) Levels of urinary NAG were measured by the synthetic substrate method (N-assay L NAG; Nittobo Co., Ltd.) Levels of urinary protein excretion, u-PCX, and urinary biomarkers were individually normalized to urinary creatinine levels.

### Statistical analysis

Continuous variables were expressed as median (interquartile range), and categorical variables were expressed as number and proportion as appropriate.

Inter-group comparisons of clinical characteristics were performed by using the Wilcoxon’s rank-sum test for continuous variables and the chi-squared test for categorical variables. Because of the highly skewed distributions of serum protein, serum albumin, serum creatinine, urinary protein, u-PCX, u-AMG, u-BMG, and u-NAG, their natural logarithms were used in subsequent analyses. P < 0.05 was considered statistically significant.

ROC analysis was performed to calculate the area under curve (AUC) into evaluating the diagnostic performance of the models. We computed the AUC with a 95% confidence interval by using 1000 bootstrap re-sampling[18]. For screening diagnostic factors in the training cohort, univariate logistic regression analysis was applied to examine the relationship between MN as a dependent variable and each of clinical parameters and biomarkers as an independent variable. Correlations between u-PCX, clinical parameters, and other urinary biomarkers were analyzed by using Pearson's correlation in the training cohort. Multivariable diagnostic models were constructed with multivariate logistic regression. We chose clinical parameters and biomarkers that had *P*-value < 0.1 on univariate logistic analysis. To choose the best diagnostic model, we selected each model based on the minimal Akaike’s information criterion (AIC).

To calculate the diagnostic score, the relative weight of a factor for predicting MN diagnosis was determined and a diagnostic score for each model was assigned by conversion of parameter estimates. Diagnostic scores were calculated 4 times for each parameter estimate, and decimals were rounded. Diagnostic scores were calculated by using the following formulas:

$$\text{Diagnostic score A }=\;\beta_{\text{PCX}}\times \text{ ln }\left(\text{u}-\text{PCX}\right)$$ (Model A)

where β is the coefficient

$$\text{Diagnostic score B }=\;\beta_{1}\times \text{ C}{\text{P}}_{1}+\;\beta_{2}\times \text{ C}{\text{P}}_{2}+\;\beta_{3}\times \text{ C}{\text{P}}_{3}+\;\ldots$$ (Model B)

where CP is the clinical parameter

$$\text{Diagnostic score C }=\;\beta_{\text{PCX}}\times \text{ ln }\left(\text{u}-\text{PCX}\right)\;+\;\beta_{1}\times \text{ C}{\text{P}}_{1}+\;\beta_{2}\times \text{ C}{\text{P}}_{2}+\;\beta_{3}\times \text{ C}{\text{P}}_{3}+\;\ldots$$ (Model C)

To evaluate the performance of the diagnostic models, we introduced a newly developed analysis technique, DCA. The method is based on the principle that the relative harms of false-positives and false-negatives can be expressed in terms of a probability threshold. The net benefit is obtained by subtracting the proportion of patients who showed false-positive results from the proportion who showed true positive results, and then weighing the relative harm of false-positive and false-negative results. The net benefit of making a decision based on the model can be calculated by using the following formula:

$$\text{Net benefit }=\frac{\text{True positives}}{\text{n}}\;–\frac{\text{Pt}}{\text{1 }-\text{ Pt}}\;\times \frac{\text{False positives}}{\text{n}}$$

where n is the total number of patients in the study and Pt is a given threshold probability.

All statistical analyses were conducted by using STATA 14.0. (Stata Corp., College Station, Texas, USA) including programs of DCA provided by Vickers[19].

## Results

### Urinary podocalyxin level and kidney diseases

**Fig 1:**
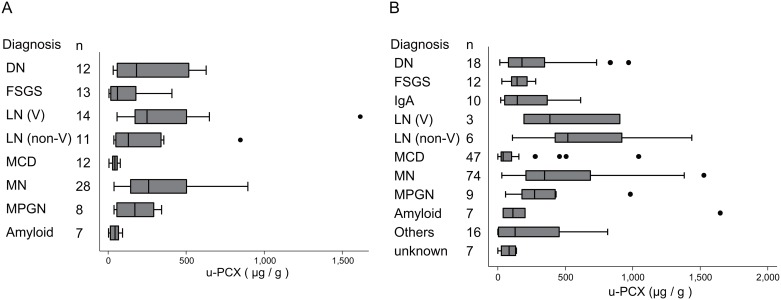
Levels of u-PCX in each kidney disease. MCD: minimal change disease; MN: membranous nephropathy; FSGS: focal segmental glomerulosclerosis; MPGN: membranoproliferative glomerulonephritis; LN: lupus nephritis; DN: diabetic nephropathy; and amyloidosis. LN was classified into two subclass; LN (V) and LN (non-V) according to the 2003 ISN/RPS classification. Class V lesion was defined as global or segmental sub-epithelial immune deposits or their morphologic sequelae, with or without class III/IV lesion. (A) Training cohort. (B) Validation cohort.

The levels of u-PCX in each disease are shown in Fig 1. Levels of u-PCX were higher among cases of DN, LN, and MN in the training cohort. The levels of u-PCX were higher among MN cases with nephrotic range proteinuria than those without. The median value of u-PCX was 399.8μg/gCr [interquartile range (IQR); 208.5–535.3] in MN cases with nephrotic–range proteinuria, whereas the median u-PCX value was 151.1μg/gCr [IQR; 113.2–244.7] in cases without nephrotic–range proteinuria (*P* = 0.026). The levels of u-PCX were also higher among cases of DN, LN, MPGN, and MN in the validation cohort. The intra- and inter-assay coefficients of variation were 4.53% and 7.12%, respectively.

### Patient characteristics

Baseline characteristics are shown in Table 1. Age was significantly higher and serum creatinine was significantly lower among MN cases.

**Table 1:** Baseline characteristics in training cohort and validation cohort.

	Training cohort (n = 105)			Validation cohort (n = 209)		
	MN (n = 41)	non-MN (n = 64)	*P* value	MN (n = 77)	non-MN (n = 132)	*P* value
Age (yr.)	64 (52, 69)	54 (39, 65.5)	0.015*	67 (61, 71)	63 (47, 71)	0.010*
Male	25 (60%)	29 (45.3%)	0.12	53 (68.8%)	97 (73.5%)	0.47
History of DM	3 (7.3%)	15 (23.4%)	0.032*	11 (14.3%)	36 (27.3%)	0.030*
BMI (kg/m^2^)	22.0 (19.5, 25.9)	23.0 (20.8, 26.2)	0.094	23.3 (21.5, 25.5)	23.0 (21, 25.9)	0.56
SBP (mmHg)	131 (121, 153)	136.5 (122, 151)	0.47	135 (125, 153)	140 (125, 154)	0.44
UPCR (g/gCr)	3.59 (2.49, 6.47)	4.78 (1.42, 7.10)	0.69	5.58 (3.39, 9.06)	5.74 (4.04, 9.44)	0.62
UACR (g/gCr)	2.59 (1.49, 4.61)	2.89 (0.75, 5.14)	0.71	3.74 (2.10, 5.18)	3.59 (2.23, 5.31)	0.95
Microscopic hematuria	16 (39%)	29 (45.3%)	0.53	40 (52.0%)	74 (56.1%)	0.57
TP (g/dL)	5.3 (4.6, 5.6)	5.4 (4.7, 6.1)	0.47	5.1 (4.6, 5.4)	5.1 (4.6, 5.7)	0.31
Alb (g/dL)	2.3 (1.9, 2.9)	2.6 (1.8, 3.1)	0.37	2.2 (1.8, 2.4)	2.1 (1.7, 2.6)	0.74
TC (mg/dL)	266 (227, 318)	260 (196, 336)	0.39	304 (253, 367)	278 (201, 390)	0.098
Cr (mg/dL)	0.8 (0.62, 1.05)	1.01 (0.73, 1.45)	0.025*	0.83 (0.7, 1.05)	1.06 (0.79, 1.61)	<0.001*
eGFR (ml/min/1.73m^2^)	70.3 (49.9, 82.2)	54.1 (35.2, 80.1)	0.061	68.8 (53, 82.2)	52.9 (32.5, 75.2)	<0.001*
ANA positive	16 (40%)	21 (32.8%)	0.46	37 (48.1%)	46 (34.9%)	0.060
u-PCX (μg/g)	254.3 (148.4, 501.6)	63.2 (39.7, 200.7)	<0.001*	365.5 (208, 687)	104.8 (34.4, 267.2)	<0.001*
u-AMG (mg/g)	21.2 (13.9, 42.4)	29.3 (12.0, 49.9)	0.93	27 (20.1, 44.4)	35.6 (20.4, 62.7)	0.54
u-BMG (μg/g)	258.8 (123.3, 1915.2)	224.5 (50.4, 3016.1)	0.38	410.5 (170.5, 1565.7)	572.3 (121.3, 6000.1)	0.070
u-NAG (IU/g)	15.7 (8.84, 32.9)	20.0 (7.71, 36.8)	0.78	25.0 (16.4, 36.9)	27.0 (15.6, 38.7)	0.73

MN = membranous nephropathy; BMI = body mass index; SBP = systolic blood pressure; UPCR = urinary protein-to-creatinine ratio; UACR = urinary albumin-to-creatinine ratio; TP = total protein; Alb = albumin; TC = total cholesterol; Cr = creatinine; eGFR = estimated glomerular filtration rate; ANA = antinuclear antibody; u-PCX = urinary podocalyxin; u-AMG = urinary α1 microglobulin; u-BMG = urinary β2 microglobulin; u-NAG = urinary N-acetyl-β-D-glucosaminidase. Continuous data represent medians (1st quartile, 3rd quartile). Categorical data indicate n values (%).

* *P* value < 0.05

The training cohort contained 41 MN cases (28 idiopathic and 14 LN with class V lesion). The validation cohort contained 77 MN cases (74 idiopathic and 3 LN with class V lesion).

### Univariate analysis and correlation analysis of u-PCX with biomarkers and clinical parameters

Univariate logistic regression analysis was applied to examine the relationship between MN as a dependent variable and each of clinical parameter and biomarker as an independent variable (S1 Table). Both continuous and categorical values of age, serum creatinine, eGFR, u-PCX, and history of diabetes mellitus (DM) were associated with diagnosis of MN.

Correlations of u-PCX with clinical parameters and other urinary biomarkers are shown in S2 Table. Urinary protein, albumin, BMG, and NAG significantly correlated with u-PCX.

### Multivariate logistic analysis for the construction of diagnostic models

We chose both continuous and categorical values of age, serum creatinine, eGFR, u-PCX, and history of DM as candidate parameters of which *P* < 0.1 in univariate analysis. Among those parameters, we constructed 4 types of diagnostic models in model B and model C (Table 2). We finally selected model B-2 and model C-2 based on a minimal AIC. To adjust the effect, age and eGFR were divided by 10, and decimals were rounded to the next integer. Diagnostic scores can be calculated by using the following formulas:

Diagnostic score A = 1 × ln(u−PCX)

$$\text{Diagnostic score B }=\;2\;\times \text{ C}{\text{P}}_{\text{age}}-\;6\;\times \text{ C}{\text{P}}_{\text{DM}}+\;1\;\times \text{ C}{\text{P}}_{\text{eGFR}}$$

CP_age_: 1 per 10 years

CP_eGFR_: 1 per 10 ml/min/1.73m^2^.

$$\text{Diagnostic score C }=\;2\;\times \text{ C}{\text{P}}_{\text{age}}-\;8\;\times \text{C}{\text{P}}_{\text{DM}}+\;1\;\times \text{C}{\text{P}}_{\text{eGFR}}+\;5\;\times \text{ ln}\left(\text{u}-\text{PCX}\right)$$

CP_DM_: 1 if patient has DM.

The above formulas were also applied for the validation cohort.

**Table 2:** Multiple models with a combination of clinical parameters and u-PCX.

		Model B				Model C			
	variables	Coefficient	95%CI	*P* value	AIC	Coefficient	95%CI	*P* value	AIC
Model 1	Age, per 10 yr.	0.42	(0.13, 0.70)	0.004	129.4	0.42	(0.10, 0.74)	0.010	103.5
	Cr, 1.0 ln mg/dl	-1.15	(-2.10, -0.20)	0.018		-1.07	(-2.13, -0.003)	0.049	
	DM	-1.49	(-2.86, -0.12)	0.033		-2.04	(-3.57, -0.52)	0.008	
	u-PCX, 1.0 ln μg/gCr	-	-	-		1.30	(0.68, 1.91)	<0.001	
Model 2	Age, per 10 yr.	0.59	(0.26, 0.91)	< 0.001	125.3	0.55	(0.20, 0.91)	0.002	100.6
	eGFR, per 10 ml/min/1.73m^2^	0.29	(0.10, 0.47)	0.002		0.27	(0.062, 0.48)	0.011	
	DM	-1.53	(-2.93, -0.13)	0.032		-2.02	(-3.55, -0.46)	0.010	
	u-PCX, 1.0 ln μg/gCr	-	-	-		1.30	(0.68, 1.93)	< 0.001	

u-PCX = urinary podocalyxin; OR = odds ratio; CI = confidence interval; AIC = Akaike's Information Criterion; AUC = area under curve; Cr = creatinine; DM = diabetes mellitus; eGFR = estimated glomerular filtration rate

**Fig 2:**
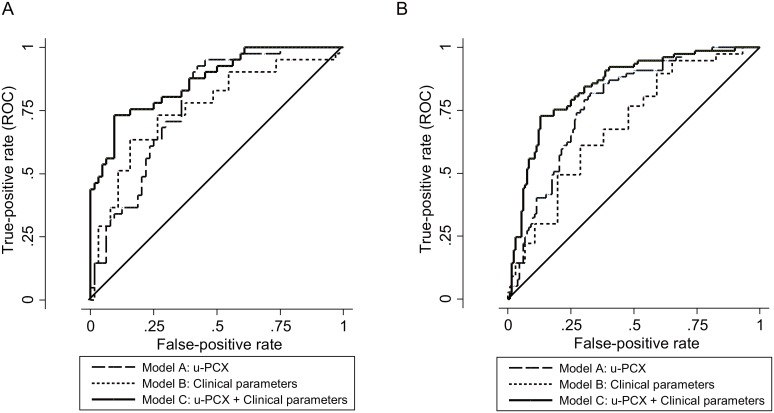
ROC curve. **(A) Training cohort.** AUC of each model is 0.777 [95% confidence interval (CI); 0.680–0.853] in Model A, 0.761 [95%CI; 0.652–0.848] in Model B, and 0.868 [95%CI; 0.781–0.931] in Model C. P value is 0.019 (A v.s. C), and 0.003 (B v.s. C). (B) Validation cohort. AUC of each model is 0.776 [95% confidence interval (CI); 0.717–0.841] in Model A, 0.690 [95%CI; 0.610–0.757] in Model B, and 0.846 [95%CI; 0.784–0.896] in Model C. P value is 0.003 (A v.s. C), and less than 0.001 (B v.s. C).

ROC curves for the training and validation cohorts are shown in Fig 2. In the training cohort, the AUC of model A, B, and C were 0.777 [95% confidence interval (CI), 0.689–0.864], 0.784 [0.691–0.878], and 0.868 [0.799–0.937], respectively. AUC of model C was significantly higher than that of models A and B (p = 0.019, 0.003, respectively). A similar result was shown in the validation cohort. The cut-off value of diagnostic score at the optimum point was 43.6 in model C. In the validation cohort, sensitivity was 80.5% and specificity was 73.5%. Cut-off points in each model are shown in S1 Dataset as a diagnostic score calculator. When you fill in the blank, diagnostic scores are calculated automatically. You can see whether the scores are higher than the cut-off points which were made in the training cohort.

### Decision curve analysis

**Fig 3:**
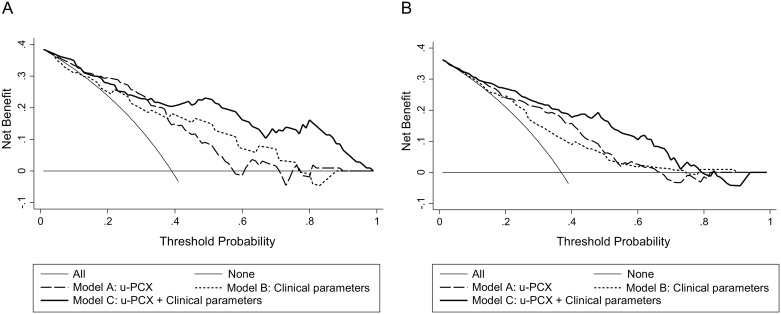
Decision curve analysis. (A) Training cohort. (B) Validation cohort.

Fig 3 illustrates the decision curves for models A, B, and C to predict the correct diagnosis of MN in patients with NS. All models were useful between threshold probabilities of 40–60%, and the net benefit of model C was better than the other 2 models between threshold probabilities of 40–80%.

## Discussion

In this study, we made 2 important clinical findings. First, u-PCX was a useful marker for the diagnosis of MN. Second, the diagnostic models combining with clinical parameters and u-PCX had clinical value. To the best of our knowledge, we are the first to develop a diagnostic model of MN using u-PCX.

The level of u-PCX was higher in MN, but in this study, u-PCX differentiated MN from NS cohort. As compared with MCD, the levels of u-PCX were higher in MN but were almost within the normal range in MCD. MCD and MN are major causes of adult onset NS; the proportion of these diseases in NS is as high as 80%, according to the Japanese epidemiological data[20].

As in the prior study, foot-process effacement, detachment from the GBM, and microvillous transformations on the apical cell surface are major processes of podocyte injuries [9]. High levels of u-PCX reflects marked microvillous transformation and vesicular shedding [21], and such phenomenon was manifested in DN, MN and LN [10]. In the case of LN, increased excretion of u-PCX was found particularly in class V of the histological subtype. The high level of u-PCX in both MN and LN with class V lesion might indicate common immunopathological mechanisms for both diseases such as complement dependent podocyte injury. Immune complexes deposited in the subepithelial region cause podocyte injury via complement activation, although the injuries do not reach cell lysis (cell death). Thus, our explanation for increased excretion of u-PCX is that the sublytic podocyte injuries activate membranous transformation, resulting in increased vesicular shedding in MN.

We then made diagnostic models for MN, combining with clinical parameters and u-PCX. When combined with clinical parameters, the diagnostic performance improved compared to the use of u-PCX only or clinical parameters only. The significant clinical parameters were age, history of DM, and renal function. MN is common in elderly patients, and renal function is prone to be relatively preserved [1]. The levels of u-PCX increased significantly in patients with MN and DN. However DN is usually clinically diagnosed, and kidney biopsy is rarely performed. Although the levels of u-PCX were also high in amyloidosis, MPGN, infection related glomerulonephritis, and lupus nephritis, they are rare diseases and have disease-specific findings such as episodes of infection, skin rash, and high titer of autoantibodies.

We performed DCA to assess performance of the diagnostic models. The net benefit of model C was better than that of the other 2 models that had threshold probabilities of 40%–80%. When we suspect that kidney biopsy may be difficult for patient, 70%–80% diagnostic probability may be sufficient to treat the patient as having MN. However in some cases, 70%–80% threshold probability is not sufficient, especially in cases with high threshold to perform immunosuppressive therapy. Therefore, for patients who have high threshold for immunosuppressive therapy, we can choose to measure more specific diagnostic markers, such as anti-PLA2R antibody. Anti-PLA2R antibody is a well-known highly specific biomarker that is used to diagnose idiopathic MN but has lower sensitivity, especially in Japan. The results of this study offered a sensitive screening marker for choosing a candidate to measure anti-PLA2R antibody.

Our study has 3 limitations. First, it is possible that the study has sampling bias. We excluded patients who received immunosuppression therapy at the time of kidney biopsy. The number of podocalyxin positive cells correlated with disease activity in patients with active glomerulonephritis [22], so we excluded such cases. In addition, 10 LN cases were excluded in the validation cohort. Second, outlier levels of u-PCX cohort can strongly affect the diagnostic scores. Among 6 LN (non-V) cases in the validation cohort, 5 patients were outliers. The diagnosis was non-V LN, but there were segmental sub-epithelial deposits in 4 out of 6 cases. Electron-dense deposits were presented patchy and segmentally in LN, so it is often difficult to detect the sub-epithelial lesion. As we mentioned above, the pathogenic mechanisms are common in LN and MN, so it may be difficult to discriminate them in the diagnostic models in the present study. However, in reality, we can differentiate them by other extra-renal manifestations before kidney biopsy. When we excluded the LN cases in analysis, AUC was slightly higher in each model; AUCs of models A, B, and C were 0.795, [95% CI; 0.732–0.857], 0.732, [0.662–0.801], and 0.862, [0.808–0.915], respectively. Finally, we did not include medical history information such as disease onset style and the duration of disease. This information is important to consider in order to make the correct diagnosis, but we did not have a clear definition.

In conclusion, we found u-PCX to be a useful biomarker for the diagnosis of MN, and we recognized the clinical utility of the diagnostic model of MN, which we made by using the combination of u-PCX and clinical parameters including age, eGFR, and history of DM. This study clearly showed the clinical significance of this diagnostic model among a population with a lower sensitivity of anti-PLA2R antibody. A prospective study is needed to improve this model, and it will need to include definitive information about the medical history.

## Supporting Information

S1 Table Univariate logistic regression analysis of clinical parameters and biomarkers to diagnose membranous nephropathy. (DOCX)

S2 Table Correlation analysis of u-PCX with clinical parameters and urinary biomarkers in the training cohort. (DOCX)

S1 Dataset Diagnostic score calculator. (XLSX)

S2 Dataset The anomymous data set of training samples. (XLSX)

S3 Dataset The anomymous data set of validation samples. (XLSX)
