# Supplementary material for: Urinary Podocalyxin as a Biomarker to Diagnose Membranous Nephropathy
Source: PLoS One. 2016 Sep 26;11(9):e0163507. doi: 10.1371/journal.pone.0163507 (PMC5036798; doi:10.1371/journal.pone.0163507)
Supplement: S1 Table — (DOCX) [file pone.0163507.s001.docx]

**S1 Table** Univariate logistic regression analysis of clinical parameters and biomarkers to diagnose membranous nephropathy

|  | OR | 95% CI | P value | AIC | AUC |
| --- | --- | --- | --- | --- | --- |
| Age | 1.03 | [1.01-1.06] | 0.018** | 138.3 | 0.641 |
| BMI | 0.94 | [0.86-1.03] | 0.18 | 142.4 | 0.597 |
| SBP | 0.99 | [0.98-1.01] | 0.48 | 142.1 | 0.542 |
| u-TP* | 1.15 | [0.81-1.64] | 0.43 | 143.8 | 0.523 |
| u-Alb* | 1.17 | [0.86-1.60] | 0.31 | 143.4 | 0.521 |
| OB ≥1+ | 0.77 | [0.35-1.71] | 0.53 | 144.1 | 0.531 |
| TP* | 0.50 | [0.07-3.77] | 0.50 | 144.0 | 0.542 |
| Alb* | 0.76 | [0.28-2.10] | 0.60 | 144.2 | 0.552 |
| Cr* | 0.42 | [0.18-0.96] | 0.040** | 139.6 | 0.630 |
| eGFR | 2.09 | [0.94-4.67] | 0.072** | 141.2 | 0.591 |
| u-PCX* | 2.95 | [1.81-4.82] | <0.001** | 116.5 | 0.777 |
| u-AMG* | 1.10 | [0.76-1.58] | 0.62 | 144.2 | 0.495 |
| u-BMG* | 1.07 | [0.92-1.25] | 0.40 | 143.8 | 0.551 |
| u-NAG* | 1.10 | [0.74-1.64] | 0.65 | 138.5 | 0.492 |
| History of　DM | 0.26 | [0.07-0.96] | 0.043** | 139.4 | 0.581 |
| ANA positive | 1.37 | [0.60-3.10] | 0.46 | 142.0 | 0.536 |

OR = odds ratio ; CI = confidence interval ; AIC = Akaike's Information Criterion ; AUC = area under curve ; BMI = body mass index ; SBP = systolic blood pressure ; u-TP = urinary total protein ; u-Alb = urinary albumin ; OB = occult blood ; TP = total protein ; Alb = albumin ; Cr = creatinine ; eGFR = estimated glomerular filtration rate ; u-PCX = urinary podocalyxin; u-AMG = urinary α1 microglobulin ; u-BMG =urinary β2 microglobulin ; u-NAG = urinary N-acetyl-β-D-glucosaminidase ; DM = diabetes mellitus ; ANA = antinuclear antibody

* Natural logarithmic values were used.

† Levels of u-PCX were categorized into less than 50, 50 or more and less than 150, 150 or more and less than 300, 300 or more.

** *P* value < 0.1
