# Supplementary material for: Urinary Podocalyxin as a Biomarker to Diagnose Membranous Nephropathy
Source: PLoS One. 2016 Sep 26;11(9):e0163507. doi: 10.1371/journal.pone.0163507 (PMC5036798; doi:10.1371/journal.pone.0163507)
Supplement: S2 Table — (DOCX) [file pone.0163507.s002.docx]

**S2 Table** Correlation analysis of u-PCX* with clinical parameters and urinary biomarkers in the training cohort

|  | r | P value |
| --- | --- | --- |
| Age | 0.096 | 0.33 |
| SBP | 0.15 | 0.12 |
| eGFR | 0.13 | 0.20 |
| TP* | -0.10 | 0.30 |
| Alb* | -0.12 | 0.22 |
| u-TP* | 0.33 | <0.001** |
| u-Alb* | 0.31 | 0.0012** |
| u-AMG* | 0.17 | 0.077 |
| u-BMG* | 0.37 | <0.001** |
| u-NAG* | 0.34 | <0.001** |

u-PCX = urinary podocalyxin; SBP = systolic blood pressure ; eGFR = estimated glomerular filtration rate ; TP = total protein ; Alb = albumin ; u-TP = urinary total protein ; u-Alb = urinary albumin ; u-AMG = urinary α1 microglobulin ; u-BMG =urinary β2 microglobulin ; u-NAG = urinary N-acetyl-β-D-glucosaminidase

* Natural logarithmic values were used.

** *P* < 0.05
